# Precuneus Dysfunction in Parkinson’s Disease With Mild Cognitive Impairment

**DOI:** 10.3389/fnagi.2018.00427

**Published:** 2019-01-10

**Authors:** Xiuqin Jia, Ying Li, Kuncheng Li, Peipeng Liang, Xiaolan Fu

**Affiliations:** ^1^State Key Laboratory of Brain and Cognitive Science, Institute of Psychology, Chinese Academy of Sciences, Beijing, China; ^2^Department of Radiology, Beijing Chaoyang Hospital, Capital Medical University, Beijing, China; ^3^Department of Radiology, Anzhen Hospital, Capital Medical University, Beijing, China; ^4^Beijing Key Lab of MRI and Brain Informatics, Beijing, China; ^5^School of Psychology, Capital Normal University, Beijing, China; ^6^Department of Psychology, University of the Chinese Academy of Sciences, Beijing, China

**Keywords:** Parkinson’s disease with mild cognitive impairment, parietal memory network, arterial spin labeling (ASL), functional connectivity (FC), precuneus

## Abstract

**Background**: Mild cognitive impairment (MCI) frequently occurs in Parkinson’s disease (PD). Neurovascular changes interact with neurodegenerative processes in PD. However, the deficits of cerebral blood flow (CBF) perfusion and the associated functional connectivity (FC) in PD patients with MCI (PD-MCI) remain unclear.

**Purpose**: This study aimed to explore the specific neurovascular perfusion alterations in PD-MCI compared to PD with normal cognition (PD-NC) and healthy controls (HCs), and to further examine the resultant whole brain FC changes in the abnormal perfusion regions.

**Methods**: Relative CBF (rCBF) was calculated using arterial spin labeling (ASL) in 54 patients with PD (27 patients with PD-NC and 27 patients with PD-MCI) and 25 HCs matched for age and gender ratio, who also underwent the structural MRI, resting-state functional MRI (rs-fMRI) and neuropsychological examinations. The gray matter (GM) changes in PD patients were analyzed using voxel-based morphometry (VBM). The alterations in rCBF perfusion and FC among groups were then analyzed respectively. Additionally, correlations between these alterations and neuropsychological performances were further examined.

**Results**: Compared to HC, left caudate atrophy was detected in patients with PD. In comparison to both PD-NC and HC, patients with PD-MCI specifically exhibited hypoperfusion in the parietal memory network (PMN) in the precuneus (PCu) and decreased PCu-FC in the right striatum. Moreover, PCu perfusion and PCu-FC strengths in the right striatum were positively associated with memory performance in PD-MCI.

**Conclusions**: These findings suggest that the posterior PMN dysfunction underlies memory deficits in PD-MCI.

## Introduction

Cognitive decline occurs commonly and frequently in patients with Parkinson’s disease (PD). Mild cognitive impairment in PD patients (PD-MCI) appears to elevate the risk for developing dementia (Janvin et al., 2006; Kehagia et al., 2010) and may even precede motor symptoms (Muslimovic et al., 2005; Aarsland et al., 2009). Examining brain function in individuals with PD-MCI may improve identification of potential markers of PD with dementia prior to the onset of significant clinical symptoms. Neurovascular dysfunction may contribute to cognitive decline in PD (Melzer et al., 2011; Fernández-Seara et al., 2012). However, little is known about the functional abnormalities that underpin the manifestation of MCI in this disease compared to PD with normal cognition (PD-NC) and healthy controls (HCs).

Arterial spin labeling (ASL) enables the quantitative measurement of cerebral blood flow (CBF; mL blood/100 g tissue/min) by using endogenous arterial blood water magnetization as a noninvasive tracer (Detre et al., 1992; Williams et al., 1992; Wolf and Detre, 2007). CBF perfusion-associated exchange of oxygen and nutrients occurring at the capillary level is considered as a close indicator of neuronal activity and glucose metabolism (Musiek et al., 2012). Abnormal CBF perfusions in the posterior cortex in PD (Melzer et al., 2011; Fernández-Seara et al., 2012) largely overlap with those reported for fluorodeoxyglucose PET metabolic deficits in this disease (Ma et al., 2007, 2010; Hosokai et al., 2009; Teune et al., 2014), and thus indicate that CBF perfusion could work as a biomarker of the neurodegenerative process in PD.

Of note, there are few CBF perfusion studies in PD-MCI. In those non-demented PD studies, the patients with PD-MCI are not differentiated from PD-NC (Fernández-Seara et al., 2012; AI-Bachari et al., 2014), which is mainly due to the lack of comprehensive definition of PD-MCI before its release by the Movement Disorder Society Task Force (Litvan et al., 2012). However, the CBF findings in PD-MCI are inconsistent and remain inconclusive. For example, posterior parieto-occipital cortex hypoperfusion in PD-MCI has been highlighted in a SPECT study in comparison to controls (Nobili et al., 2009). In another study, CBF perfusion has been investigated in PD with the entire spectrum of cognitive status (including NC, MCI, and dementia) using ASL, which therefore fails to identify the alteration in CBF perfusion specific to PD-MCI (Melzer et al., 2011). On the other hand, studies with non-demented PD (combined with part of patients with MCI) exhibited either subcortical hypoperfusion and widespread cortical hypoperfusion in the posterior parieto-occipital cortex, precuneus (PCu)/cuneus, and frontal cortex coupled with some of them being atrophied (Fernández-Seara et al., 2012), or failed to find any group difference in non-demented PD compared to HC (AI-Bachari et al., 2014). These inconsistencies may result from the heterogeneity in PD-MCI samples and the confounding effect (e.g., ON medication or OFF medication) on results between group comparisons. Accordingly, the specific perfusion alteration in well-defined PD-MCI has not been fully described and remains unclear in comparison with PD-NC and HCs. On the basis of previous studies (Nobili et al., 2009; Fernández-Seara et al., 2012), it was hypothesized that the PD-MCI might exhibit hypoperfusion in the parietal-occipital network.

Moreover, it has been previously proposed that the alteration in neurovascular perfusion significantly affects the blood oxygenation level-dependent (BOLD) signal (D’Esposito et al., 2003) and is associated with functional dysconnectivity (Yoshiura et al., 2009). Low-frequency BOLD signal in resting-state functional MRI (rs-fMRI) provides a sensitive measure of brain activity. Several previous resting-state FC (rs-FC) studies have addressed functional alterations in PD-MCI. These studies have demonstrated that PD-MCI exhibits FC alterations mainly in the dorsal attention network (DAN), default mode network (DMN) and fronto-parietal network (FPN) in PD-MCI (Amboni et al., 2015; Baggio et al., 2015; Chen et al., 2017; Bezdicek et al., 2018). However, how the altered neurovascular perfusion interacts with the disrupted FC remains still unclear. To capture the subtle neural changes occurring in PD-MCI, seed-to-voxel FC, an approach to assess functional integrity among structurally segregated brain regions, was further conducted based on abnormal CBF perfusion regions in PD-MCI.

Hence, the present study first aimed to examine the alterations of CBF perfusion specific to PD-MCI compared to PD-NC and HC. Based on previous findings that CBF alterations occur gradually in PD patients with cognitive progression (Melzer et al., 2011) and the posterior cortical hypoperfusion is related to cognitive decline in non-demented PD (Syrimi et al., 2017), it was thus hypothesized that PD-MCI might exhibit abnormal CBF perfusion in posterior parietal cortical regions. Second, the present study aimed to explore the changes of neural activity by FC in the altered perfusion regions in PD-MCI compared to PD-NC and HC. It was further hypothesized that functional dysconnectivity may appear in PD-MCI in regions with altered CBF perfusion in this disease. Finally, the present study aimed to reveal how these brain degenerations associate with neuropsychological deficits in PD-MCI.

## Materials and Methods

### Subjects

In the present study, 60 patients with PD (30 for PD-NC and 30 for PD-MCI) who met the UK Bank criteria for the diagnosis of PD (Hughes et al., 1992) were recruited in the present study. All PD participants were examined in their OFF medication state, that is, at least after a 12-h withdrawal of anti-Parkinson medication. The HC group comprised 30 volunteers who were matched for age and gender ratio. Six patients and five healthy volunteers were excluded from the study due to motion artifacts and low quality of MRI data. At last, 27 PD-NC (15 males, 63.11 ± 9.27 years old), 27 PD-MCI (16 males, 62.59 ± 6.61 years old), and 25 HCs (11 males, 59.44 ± 5.77 years old) were included in this study. None of the participants suffer from depression, determined with the 17-item Hamilton Depression Rating Scale (HAMD-17; Hamilton, 1960). Patients with MCI were defined by Level II of the Movement Disorder Society Task Force (Litvan et al., 2012). The study was approved by the Research Ethics Committee of Xuanwu Hospital, Capital Medical University and written informed consent was obtained from each participant.

### Diagnostic Criteria and Neuropsychological Assessment

For the clinical characteristics, disease stage was scored using the H&Y state score and disease severity was captured by the UPDRS part III. The neuropsychological tests that included two tests within each of the five cognitive domains were as follows: (i) Trait Making Test and Digit Span Test within attention and working memory domain; (ii) Verbal Fluency Test and Clock Drawing Test within executive domain; (iii) Wechsler Adult Intelligence Scale-IV (WAIS-IV) and Boston Naming Test within language domain; (iv) Rey’s Auditory Verbal Learning Test (AVLT) and Logical Memory subset within memory domain; and (v) Benton’s Judgment of Line Orientation (JOLO) and Clock copying within visual spatial function domain.

On the basis of comprehensive neuropsychological tests, patients with PD were classified as cognitively normal and with MCI. The cognitive impairment in PD was defined based on the detailed cognitive battery: expected z-scores for each test and each subject were calculated based on a multiple regression analysis performed in the HC group adjusted for age, gender, and education (Aarsland et al., 2009). A subject was defined as MCI when the actual z-score for a given test was at least 1.5 SD lower than the expected score in at least two tests in one domain or in at least one test in at least two domains.

### MRI Data Acquisition

MRI data were acquired using a Siemens Tim Trio 3T MRI system (Siemens, Erlangen, Germany) by using an 8-channel heal coil. Foam padding and headphones were used to limit head motion and reduce scanner noise. Participants were instructed to keep still and remain motionless. High-resolution structural images were acquired by using 3D T1-weighted magnetization-prepared rapid gradient echo (MPRAGE). Scan parameters were as follows: TR = 1,900 ms, TE = 2.22 ms, flip angle = 9°, matrix size = 256 × 256, 176 1 mm saggital slices. Resting-state pulsed ASL (pASL) perfusion images were collected using Q2TIPS II with the following parameters: labeling time = 700 ms, post-labeling delay = 1,100 ms, slice time = 41 ms, TR = 3,000 ms, TE = 11 ms, field of view (FOV) = 192 mm × 192 mm, matrix = 64 × 64, flip angle = 90°, 25 axial slices, and slice thickness = 4 mm. One-hundred and one ASL images were acquired from each subject and the first one was taken as the equilibrium magnetization of the brain (M0) image for CBF calculation. rs-fMRI data were obtained by using an echo-planar imaging sequence that lasted 8 min (240 volumes) with the following parameters: TR = 2,000 ms, TE = 30 ms, flip angle = 90°, FOV = 240 mm × 240 mm, matrix size = 64 × 64, 33 slices, and slice thickness = 4 mm.

### Data Preprocessing

#### Voxel-Based Morphometry Analysis

To determine the structural abnormalities in patients, we performed a voxel-based morphometry (VBM) analysis for all structural images of PD-NC, PD-MCI, and HC using SPM12. T1-weighted images were segmented using the unified segmentation model into gray matter (GM), white matter (WM), and cerebrospinal fluid (CSF) based on tissue probability maps (TPMs) in Chinese2020 space^1^ (Liang et al., 2015; Shi et al., 2017). Non-linear warping of GM images was then performed on the GM template in Chinese2020 space. The spatially normalized GM maps were modulated by the Jacobian determinant of the deformation field and corrected for individual brain sizes. The modulated, normalized GM images (voxel size 1 × 1 × 1 mm^3^) were smoothed with a 6-mm full width at half maximum (FWHM) isotropic Gaussian kernel.

#### ASL Data Analysis

ASL data were preprocessed using SPM12^2^ based on batch scripts available in ASLtbx (Wang et al., 2008). The following processing was applied: (i) the structural and ASL images were first reoriented to the center of the image matrix; (ii) ASL image pairs were realigned and corrected for head motion (Wang, 2012); (iii) ASL images were then co-registered to their T1 structural images; (iv) high-pass filtering (cutoff = 0.5) was performed for temporal ASL image denoising; (v) Gaussian kernel (FWHM = 6 mm) was then applied on the ASL images; (vi) after regressing out residual motions and global signal from ASL images, CBF images were then calculated according to Wang et al. (2005); (vii) T1 images were segmented to GM, WM, and CSF by normalizing into Chinese2020 TMPs and resampled them to 3 × 3 × 3 mm^3^; and (viii) the mean relative CBF (rCBF) images which were spatially normalized into Chinese2020 template space by using the transformations from segmentation in step (vii) were used to eliminate the global CBF effect.

#### Rs-fMRI Preprocessing and Functional Connectivity

Rs-fMRI data were preprocessed using SPM12 and seed-to-voxel correlation analysis was carried out by the FC (CONN) toolbox v17c (Whitfield-Gabrieli and Nieto-Castanon, 2012). The first 10 functional images were discarded to reduce the fluctuation of the MRI signal in the initial stage of scanning. The remaining 230 images of each subject were first corrected for slice timing to reduce the within-scan acquisition time differences between slices and then realigned to eliminate the influence of head motion during the experiment. All subjects included in the present study exhibited head motion less than 1.5 mm in any of the *x, y*, or *z* directions and less than 1.5° of any angular dimension. Next, the realigned images were co-registered to T1 images. Then, T1 images were segmented into GM/WM/CSF by using Chinese2020 TMPs. Then the co-registered images were spatially normalized into Chinese2020 template space using transformations from segmentation and resampled them to 3 × 3 × 3 mm^3^. Subsequently, the functional images were smoothed with a 6-mm FWHM isotropic Gaussian kernel. After preprocessing, images were then band-pass filtered to 0.008–0.09 Hz to reduce the influence of noise. Further denoising steps included regression of the six motion parameters and their first-order derivatives, regression of WM and CSF signals following the implemented CompCor strategy (Behzadi et al., 2007) and a linear detrending. The seed regions were determined by the areas that showed significant rCBF alterations specific to patients with PD-MCI. The correlation coefficients between the seed voxels and all other brain voxels were computed to generate correlation maps. For group analyses the correlation coefficients were converted to *z*-values using Fisher’s *r*-to-*z* transformation to improve normality (Lowe et al., 1998).

### Statistical Analysis

As to the clinical and neuropsychological measurements, the normality of clinical data and neuropsychological measures was firstly evaluated by a Kolmogorov-Smirnov (KS) test in order to choose parametric tests or non-parametric tests using SPSS 22. Independent one-way analysis of variance (ANOVA; for parametric test) or Kruskal-Wallis testing (for non-parametric test) was performed for comparing the three groups, with Bonferroni correction used for the *post hoc* comparisons between groups (Student *t*-test for parametric testing and Mann-Whitney for non-parametric testing).

The rCBF maps, seed-voxel FC maps, and GM maps were analyzed using General Linear Model on a voxel-wise comparison across the whole brain. An absolute GM threshold of 0.2 was used to avoid possible edge effects around the border between GM and WM. Alterations in GM volume, rCBF, and seed-to-voxel FC among the three groups were assessed using one-way analysis of covariance (ANCOVA) controlled for nuisance variables. The demographic features that showed significant group differences except for the cognitive profiles were taken as nuisance variables of no interest. Furthermore, the mean frame-wise displacement (FD; Power et al., 2015) and global correlation (GCOR; Saad et al., 2013), which represented the average correlation coefficient between every pair of voxels across the entire brain, were included as nuisance variables to further exclude the influence of head motion and global signal in seed-voxel FC analysis. *Post hoc* two-sample *t*-tests were then conducted among HC, PD-NC, and PD-MCI masking ANCOVA results inclusively.

GM volume and FC results were reported based on an uncorrected voxel-wise height threshold of *p* < 0.001 combined with an FWE-corrected cluster-wise threshold of *p* < 0.05. Due to our* a priori* hypothesis about the localization of perfusion changes, significance level was set at voxel-wise *p* < 0.001 uncorrected for CBF analysis. Then a small volume correction (SVC) was applied to investigate which subregions in the posterior parietal and occipital areas might be more affected in PD-MCI patients and results were thresholded at FWE *p* < 0.05. Brain region was localized with xjView^3^.

### Correlation Analysis

In addition, alterations in rCBF perfusion and FC specific to PD-MCI were defined as regions of interest (ROIs). Partial correlation analyses were further examined between these alterations and neuropsychological performances that exhibited significant differences between PD groups controlled for nuisance variables, similar to the corresponding CBF or FC analyses (*p* < 0.05 Bonferroni correction).

## Results

### Demographic Results

As shown in Table 1, ANOVA analyses showed that significant differences were found in education level (*p* < 0.001) and HAMD (*p* < 0.01). *Post hoc* comparisons revealed that significant lower education level was detected in PD-MCI compared to HC and PD-NC, and significant higher HAMD scores were detected in PD-MCI and PD-NC compared to HC. As to the five cognitive domains, individuals with PD-MCI exhibited significant cognitive dysfunction in all five cognitive domains compared to HC and PD-NC, with the exception of no significant differences between PD-MCI and PD-NC in verbal fluency tests and AVLT delayed recall, while PD-NC patients only exhibited cognitive impairment in the verbal fluency test compared to HC (Table 1). There was no other significant difference in the remaining comparisons.

**Table 1:** Demographic and clinical characteristics.

	HC (*n* = 25)	PD-NC (*n* = 27)	PD-MCI (*n* = 27)	*p*-value
Age (years)^#^	59.44 (5.77)	63.11 (9.27)	62.59 (6.61)	0.17
Gender (male/female)	11/14	15/12	16/11	0.61
Education (years)	12.08 (2.91)	13.37 (2.91)	7.78 (4.27)	<0.001^†‡^
Disease duration (years)	—	3.71 (2.96)	3.65 (2.86)	0.82
Hoehn and Yahr stage	—	1.88 (0.50)	1.71 (0.68)	0.20
UPDRS Part III^#^	—	23.82 (7.38)	21.38 (9.53)	0.38
L-DOPA dose (mg/day)	—	334.13 (255.73)	370.88 (392.0)	0.93
HAMD	1.80 (2.29)	2.89 (1.50)	3.44 (2.31)	0.006*^†^
Attention and working memory				
Trait making test (B-A)	30.52 (11.97)	37.22 (26.09)	99.78 (57.97)	<0.001^†‡^
Digit span forward	7.20 (1.32)	7.22 (1.55)	5.63 (1.45)	<0.001^†‡^
Digit span backward	5.44 (1.56)	4.63 (1.33)	3.52 (0.98)	<0.001^†‡^
Executive function				
Verbal fluency test	21.80 (4.00)	17.04 (3.24)	15.44 (4.29)	<0.001*^†^
Clock drawing test	9.52 (0.92)	9.52 (1.19)	7.07 (2.53)	<0.001^†‡^
Language				
WAIS-IV	17.44 (2.75)	17.19 (3.49)	9.48 (3.66)	<0.001^†‡^
Boston naming test	24.76 (1.79)	24.07 (2.99)	19.93 (4.44)	<0.001^†‡^
Memory				
AVLT immediate recall^#^	29.56 (6.64)	26.96 (5.91)	21.89 (4.60)	<0.001^†‡^
AVLT delayed recall	10.40 (2.60)	9.44 (2.38)	7.56 (3.70)	<0.001^†^
Logic memory immediate recall	5.20 (1.91)	5.19 (1.39)	2.96 (1.76)	<0.001^†‡^
Logic memory delayed recall	4.29 (1.40)	4.15 (1.63)	2.26 (1.77)	<0.001^†‡^
Visual spatial function				
CLOX	14.04 (0.73)	13.39 (1.11)	11.37 (2.18)	<0.001^†‡^
JOLO	21.36 (5.15)	19.78 (4.79)	14.78 (5.61)	<0.001^†‡^

Note: data are expressed as mean (standard deviation). Gender data were analyzed with χ^2^ test. Other *p* values were derived from Kruskal Wallis test or Mann-Whitney test except for ^#^ that was derived from the independent one-way analysis of variance (ANOVA) or two sample *t*-test. *Post hoc comparisons showed significant differences between healthy controls (HCs) and patients with Parkinson’s disease with normal cognition (PD-NC); ^†^*post hoc* comparisons showed significant differences between HCs and patients with PD-mild cognitive impairment (PD-MCI); ^‡^*post hoc* comparisons showed significant differences between patients with PD-NC and those with PD-MCI. UPDRS, unified Parkinson’s disease rating scale; HAMD, Hamilton depression rating scale; JOLO, Benton Judgment of Line Orientation.

### VBM Results

Significant GM atrophy was commonly detected in the left caudate (MNI: −6, 7, 5; Chinese2020: −6, 7, 4 with 89 voxels) in patients with PD-NC and PD-MCI compared to HC. No significant difference was detected between PD-NC and PD-MCI.

### RCBF Results

**Figure 1:**
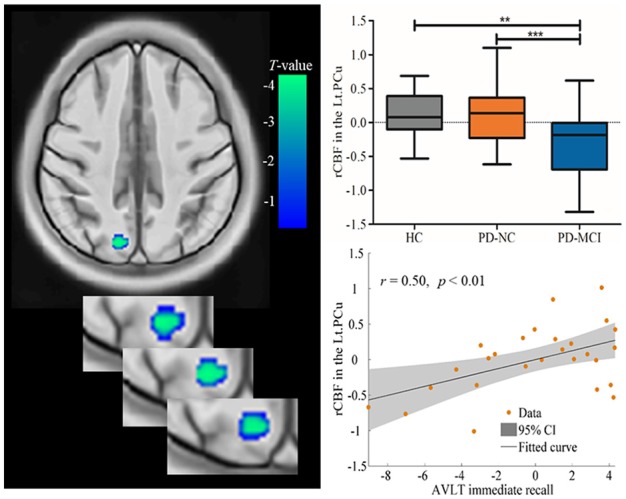
Decreased rCBF values in the Lt.PCu specific to PD-MCI compared to HC and PD-NC. Box plots with Whiskers min to max show the rCBF values in the Lt.PCu in the three groups and scatterplots show the relationship between the Auditory Verbal Learning Test (AVLT) immediate recall performance and rCBF values in the Lt.PCu in patients with PD-MCI after controlling for education and depression score. rCBF, relative cerebral blood flow; Lt, left; PCu, precuneus; HC, healthy control; PD-NC, Parkinson’s disease with normal cognition; PD-MCI, Parkinson’s disease with mild cognitive impairment. **p < 0.01; ***p < 0.001.

Significant hypoperfusion in the left PCu was detected in ANCOVA analysis, controlled for education level, and HAMD scores. *Post hoc* two-sample *t*-testing further revealed that the hypoperfusion in the left PCu was specific to PD-MCI when compared to PD-NC and HC (Table 2, Figure 1). The region that exhibited hypoperfusion in PD-MCI common to PD-NC was defined as the seed region (MNI: −14, −68, 38 with 111 voxels; *t* = 3.96) in the FC analysis.

**Table 2:** Relative CBF (rCBF) changes among groups of HC, PD-NC, and PD-MCI.

Region	Cluster size	MNI	Chinese2020	*t*-value
	(voxel)	(*x, y, z*)	(*x, y, z*)	
HC>PD-MCI				
Lt.PCu	118	(−14, −68, 38)	(−14, −59, 39)	3.96
PD-NC>PD-MCI				
Lt.PCu	194	(−15, −69, 36)	(−15, −60, 38)	4.30

*Note: PCu, precuneus; Lt, left*.

In addition, correlation analysis found that the rCBF values in the left PCu region were positively associated with AVLT immediate recall (*r* = 0.50, *p* < 0.01), controlled for education level and HAMD scores (see Figure 1).

### FC Results

**Figure 2:**
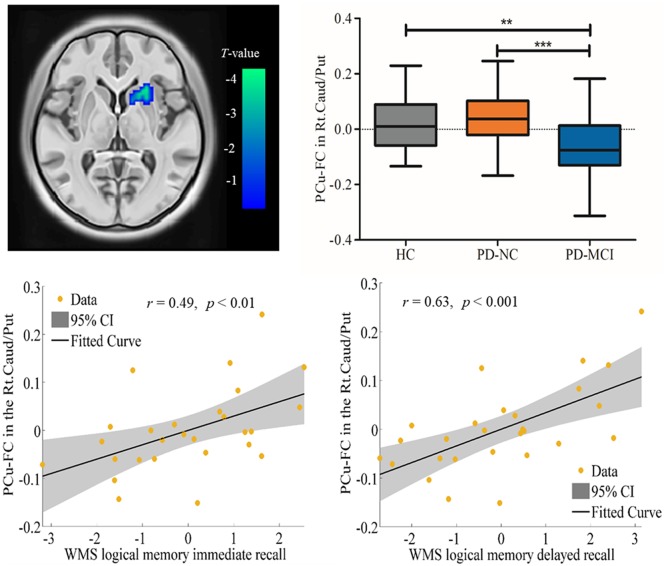
Decreased functional connectivity (FC) in the seed region of the Lt.PCu in the Rt.Caud/Put specific to PD-MCI compared to HC and PD-NC. Box plots with whiskers min to max show the FC values between the Lt.PCu and the Rt.Caud/Put in the three groups and scatterplots show the relationship between the FC strength in the two regions and WMS logical memory performances in patients with PD-MCI after controlling for education and depression score. Lt, left; Rt, right; PCu, precuneus; Caud, caudate; Put, putamen; HC, healthy control; PD-NC, Parkinson’s disease with normal cognition; PD-MCI, Parkinson’s disease with mild cognitive impairment. **p < 0.01; ***p < 0.001.

Spontaneous brain neural activity that is associated with neurovascular perfusion and hypoperfusion leads to disrupted FC. In the present study, the identified hypoperfusion in the PCu was further taken as seed regions for FC analysis. The FC results showed that significant decreased FC was detected in the right caudate/putamen in PD-MCI when compared to HC (MNI: 21, 18, 0 with 33 voxels; *t* = 4.96) and PD-NC (MNI: 24, 12, −3 with 46 voxels; *t* = 4.47; see Table 3, Figure 2). Correlational analysis further revealed that the FC between the seed PCu and the right caudate/putamen was positively correlated with WMS logical memory immediate recall (*r* = 0.49, *p* < 0.01) and delayed recall (*r* = 0.63, *p* < 0.001) performances controlled for the education level, and HAMD scores (see Figure 2).

**Table 3:** Decreased functional connectivity (FC) in seed region of the Lt.PCu in PD-MCI compared to HC and PD-NC.

Region	Cluster size	MNI	Chinese2020	*T*-value
	(voxel)	(*x, y, z*)	(*x, y, z*)	
HC>PD-MCI				
Rt.Caudate/Putamen	33	(21, 18, 0)	(20, 16, −1)	4.96
PD-NC>PD-MCI				
Rt.Caudate/Putamen	46	(24, 12, −3)	(23, 11, −3)	4.47

*Note: Rt, right*.

## Discussion

The current study investigated the specific alteration pattern of CBF perfusion in PD-MCI in comparison to both PD-NC and HC, and further investigated how neurovascular dysfunction affects neural activity in PD-MCI. Specifically, the PCu hypoperfusion was identified in PD-MCI, and the reduced PCu-FC in the striatum was further detected specific to this disease. Moreover, the PCu dysfunctions (i.e., hypoperfusion and disrupted FC) were positively correlated with memory performance in PD-MCI, which suggests that malfunction (neurovascular and neural functional activity) in the posteromedial parietal region may contribute to memory decline within individuals with PD-MCI.

### GM Atrophy in PD

In line with previous PD studies (Brenneis et al., 2003; Lee et al., 2011), in the present study significant GM atrophy was detected in the left caudate in PD patients (independently of the cognitive status). Previous studies have reported GM atrophy in PD-MCI but still lack consistency. For example, Chen et al. (2017) reported cortical atrophy in frontal and temporal areas in PD-MCI, but Hattori et al. (2012) failed to detect any difference between PD-MCI and PD-NC or HC. These inconsistences might be due to the heterogeneities of patients, such as different stages of disease and medication-related confounding.

### Hypoperfusion in the PCu Specific to PD-MCI

In line with a previous SPECT study (Nobili et al., 2009), posterior cortical hypoperfusion, particularly in the PCu, was found specific to PD-MCI. Recently, studies have indicated that the PCu is a key node of the “parietal memory network” (PMN), which is recruited in memory encoding and retrieval tasks (Gilmore et al., 2015; Hu et al., 2016). Furthermore, in the current study, it was revealed that the PCu perfusion positively correlated to memory function measured by AVLT immediate recall, which implies the neural characteristics of memory deficits in PD-MCI. To date, no ASL study has characterized the CBF perfusion pattern specific to well-defined PD-MCI patients in comparison to PD-NC and HC. Recently, the only ASL study including PD-MCI found decreased perfusion in individuals with PD in full range of cognitive status compared to controls, whereas specific perfusion characteristics in PD-MCI were not specified as no *post hoc* comparison was performed after ANOVA (Melzer et al., 2011). In addition, previous morphometry studies have revealed GM atrophy in PD patients even at early stage (Hong et al., 2012; Lee et al., 2013; Jia et al., 2015), which may interact with cortical perfusion deficits (Fernández-Seara et al., 2012).

More recently, a study in individuals with non-demented PD (combined PD-NC and PD-MCI) found that CBF abnormalities in the PCu are associated with global cognitive deficits (Syrimi et al., 2017). However, the present study did not detect significant differences between PD-NC and HC. The following explanations may account for the inconsistency. One possibility was that in the present study the cognitive status in PD patients was comprehensively examined including five cognitive domains and was well-matched between PD-NC and HC; while in the other study cognitive status was tested by a global cognitive assessment, therefore it lacked a detailed neuropsychological examination in non-demented PD. In any case, the cognitive status in controls was not available. Furthermore, the PD patients in the present study were examined in their OFF medication state, while in that study PD participants were tested in their ON medication state, which could induce CBF changes in PD (Kobari et al., 1995). Taken together, the current findings suggested that PCu hypoperfusion might be a specific characteristic of PD-MCI, which further differentiates the neural underpinnings of cognitive decline in PD-MCI from non-demented PD.

### Decreased PCu-FC in the Striatum

Neurovascular function is tightly coupled with neural activity measured by BOLD signal. In the present study, the reduced PCu FC in the hypoperfusion region with the striatum (caudate/putamen) was detected in PD-MCI, controlling for the potential effect of brain structural alteration. Indeed, the striatum and PCu have been reported to be anatomically and functionally connected (Selemon and Goldman-Rakic, 1985; Cavanna and Trimble, 2006), which implies the abnormal perfusion in the PCu in PD-MCI afflicts its functional synchronization with the striatum. It is noteworthy that the loss of dopaminergic neurons in the nigrostriatal pathway results in the depletion of dopamine in the striatum (Ravina et al., 2012; Buddhala et al., 2015). Specifically, alteration of the striatum in PD has been consistently reported in non-demented PD both structurally (Kostic et al., 2012; Jia et al., 2015) and functionally (Shin et al., 2016). In addition, a previous study has indicated that older adults recruit the opposing homogenous hemispheric regions to maintain task performance (Cabeza, 2002). In the present study, the decreased PCu-FC in the contralateral striatum in patients with PD-MCI may imply a damaged ability to recruit interhemispheric FC for compensation for age-related declines in the structural integrity of long intrahemispheric connections (Hermundstad et al., 2013). The positive association between the PCu-FC in the striatum and memory performance measured by logic memory in PD-MCI, further supports the predictive potential of PCu degeneration on memory dysfunction in this disease.

However, a limitation should be recognized in the current study. Besides ASL, WM status could also reflect neurovascular integrity. A recent study has reported significant DTI changes in PD that precede GM changes (Rektor et al., 2018). Further studies need to explore neurovascular integrity considering the WM alteration revealed by DTI data. Overall, the current findings of PCu abnormality (i.e., hypoperfusion and abnormal FC) specific to PD-MCI, suggest that combination between CBF and BOLD could provide insight into the mechanisms underlying cognitive decline in PD-MCI.

## Author Contributions

KL, PL, and XF contributed to the study conception and design. YL carried out data collection. Data were analyzed and interpreted by XJ. The drafting of manuscript was written by XJ and was critically revised by PL and XF. The final approval of the version to be published was confirmed by PL, XF and XJ.

## Conflict of Interest Statement

The authors declare that the research was conducted in the absence of any commercial or financial relationships that could be construed as a potential conflict of interest.

## Footnotes

^1^http://www.chinese-brain-atlases.org

^2^http://www.fil.ion.ucl.ac.uk/spm

^3^http://www.alivelearn.net/xjview
